# The bidirectional relationship between epilepsy and sleep: a narrative review

**DOI:** 10.3389/fneur.2026.1882139

**Published:** 2026-09-17

**Authors:** Giulia Bellone, Martina Bonometti, Alice Rubeo, Melania Evangelisti, Sara Mascambroni, Giovanni Di Nardo, Maurizio Mennini, Pasquale Parisi, Alessandro Ferretti

**Affiliations:** 1 Pediatrics Unit, Department of Neuroscience, Mental Health and Sense Organs (NESMOS), Faculty of Medicine and Psychology, Sant'Andrea Hospital, Sapienza University of Rome, Rome, Italy; 2 Pediatric Gastroenterology and Endoscopy Unit, Department of Pediatric Specialties, Santobono Pausilipon Children’s Hospital, Naples, Italy

**Keywords:** antiseizure medications, epilepsy, narrative review, obstructive sleep apnea, parasomnia, sleep architecture, sleep disturbance

## Abstract

**Background:**

Epilepsy and sleep disorders are highly prevalent conditions that frequently coexist, exerting reciprocal adverse effects through shared neurobiological mechanisms.

**Objective:**

We conducted a narrative review of the literature addressing the bidirectional relationship between epilepsy and sleep disorders, aiming to provide a comprehensive synthesis of its neurophysiological basis, clinical implications, and therapeutic consequences.

**Results:**

The epilepsy–sleep relationship is mediated by multiple neurobiological mechanisms, including thalamocortical network dynamics, circadian clock gene regulation, adenosinergic and orexinergic tone, synaptic homeostasis, and glymphatic clearance. A key pathophysiological concept underlying this relationship is that NREM sleep promotes interictal epileptiform discharges and seizure propagation through thalamocortical synchronization, whereas REM sleep exerts a protective effect. Epileptic activity disrupts both sleep macro- and microarchitecture, reducing total sleep time, sleep efficiency, and REM sleep, increasing wake after sleep onset, and destabilizing NREM microarchitecture as reflected by altered cyclic alternating pattern rates. Conversely, sleep disturbances lower the seizure threshold and contribute to disease progression: sleep deprivation is an independent seizure-precipitating factor, and comorbid obstructive sleep apnea further lowers the seizure threshold through intermittent hypoxia and arousal instability. Specific epileptic syndromes with a privileged sleep relationship are also reviewed, including sleep-related hypermotor epilepsy, epileptic encephalopathy with spike–wave activation in sleep, and self-limited childhood focal epilepsies. The differential diagnosis between NREM parasomnias and sleep-related epilepsies remains challenging; the diagnostic workup should always include a thorough clinical history characterizing the episodes, followed by video-polysomnography, which represents the gold standard. From a therapeutic standpoint, antiseizure medications exert heterogeneous effects on sleep architecture, and polytherapy is independently associated with worse sleep quality; targeting sleep disturbances therefore represents a modifiable strategy capable of improving seizure control, cognitive function, and quality of life.

**Conclusion:**

A multidisciplinary approach integrating systematic screening for sleep disorders, optimization of antiseizure medication timing and selection, treatment of comorbid obstructive sleep apnea, and behavioral sleep interventions has the potential to meaningfully improve outcomes in patients with epilepsy. Wider implementation of these strategies in routine clinical practice is both a priority and an opportunity.

## 1 Introduction

Epilepsy and sleep disorders are highly prevalent conditions in the general population and frequently coexist in clinical practice (1). Epilepsy is defined by at least two unprovoked seizures occurring more than 24 h apart, a single unprovoked seizure with ≥60% recurrence risk, or the diagnosis of a defined epileptic syndrome (2). It is among the most common neurological disorders worldwide, with a prevalence of about 6.4 per 1,000 individuals and an incidence rate of 61.4 per 100,000 person-years (3, 4). Sleep disorders represent a growing public health concern (5) and are classified by the International Classification of Sleep Disorders, Third Edition (ICSD-3) into seven categories: insomnia, sleep-related breathing disorders, central hypersomnolence disorders, circadian rhythm disorders, sleep-related movement disorders, parasomnias, and other sleep disorders (6). The association between epilepsy and sleep disorders is well established, with sleep disorders being significantly more prevalent in patients with epilepsy than in the general population (7, 8). In adults with epilepsy, sleep disorders are up to three times more frequent, and in children up to ten times more frequent than in healthy peers (8, 9). They are linked to poorer seizure control and reduced quality of life, representing modifiable factors for improving outcomes (10). The bidirectional relationship between epilepsy and sleep has been investigated since the late nineteenth century. In 1885, Gowers classified seizure timing as diurnal, nocturnal, or diffuse, providing early evidence of an interaction with the sleep–wake cycle. Later, the introduction of electroencephalography (EEG) and polysomnography (PSG) enabled systematic investigation, highlighting a complex, multifactorial relationship. Recognizing this bidirectionality is essential for education and treatment optimization (11). Improving sleep quality and treating sleep disorders are associated with better seizure control and well-being, suggesting a potential disease-modifying therapeutic strategy (10).

## 2 Objectives of the narrative review: rationale for studying the bidirectional relationship

Epilepsy and sleep disorders frequently coexist, reflecting a close and complex relationship mediated by shared neurobiological mechanisms. As illustrated in Figure 1, this relationship is inherently bidirectional. Across sleep stages (NREM and REM), patterns of neuronal synchronization and desynchronization influence epileptogenic discharges and seizure characteristics, including type and frequency. Conversely, disruption of sleep structure and stage progression contributes to seizure worsening through cortical hyperexcitability, with sleep deprivation being a common precipitating factor. Epileptic activity, both ictal and interictal, and recurrent seizures affect sleep continuity and both micro- and macro-architecture. After a seizure, whether symptomatic or not, an increase in wake time after sleep onset has been described (12). Studies also show reduced REM sleep, delayed REM onset, and impaired NREM stability due to epileptic discharges (13). Sleep plays a critical role in cognitive development and memory consolidation; alterations in sleep structure and cyclicity in pediatric epilepsy populations impair memory consolidation processes (14). This relationship has therapeutic implications: antiseizure medications (ASMs) may alter sleep architecture independently of seizure control, while some sleep treatments may reduce seizure frequency (15). This narrative literature review aims to provide an in-depth and detailed examination of the bidirectional relationship between epilepsy and sleep disorders, with the goal of optimizing seizure control, improving sleep architecture and quality, and consequently promoting a significant enhancement in patients’ quality of life.

**Figure 1:**
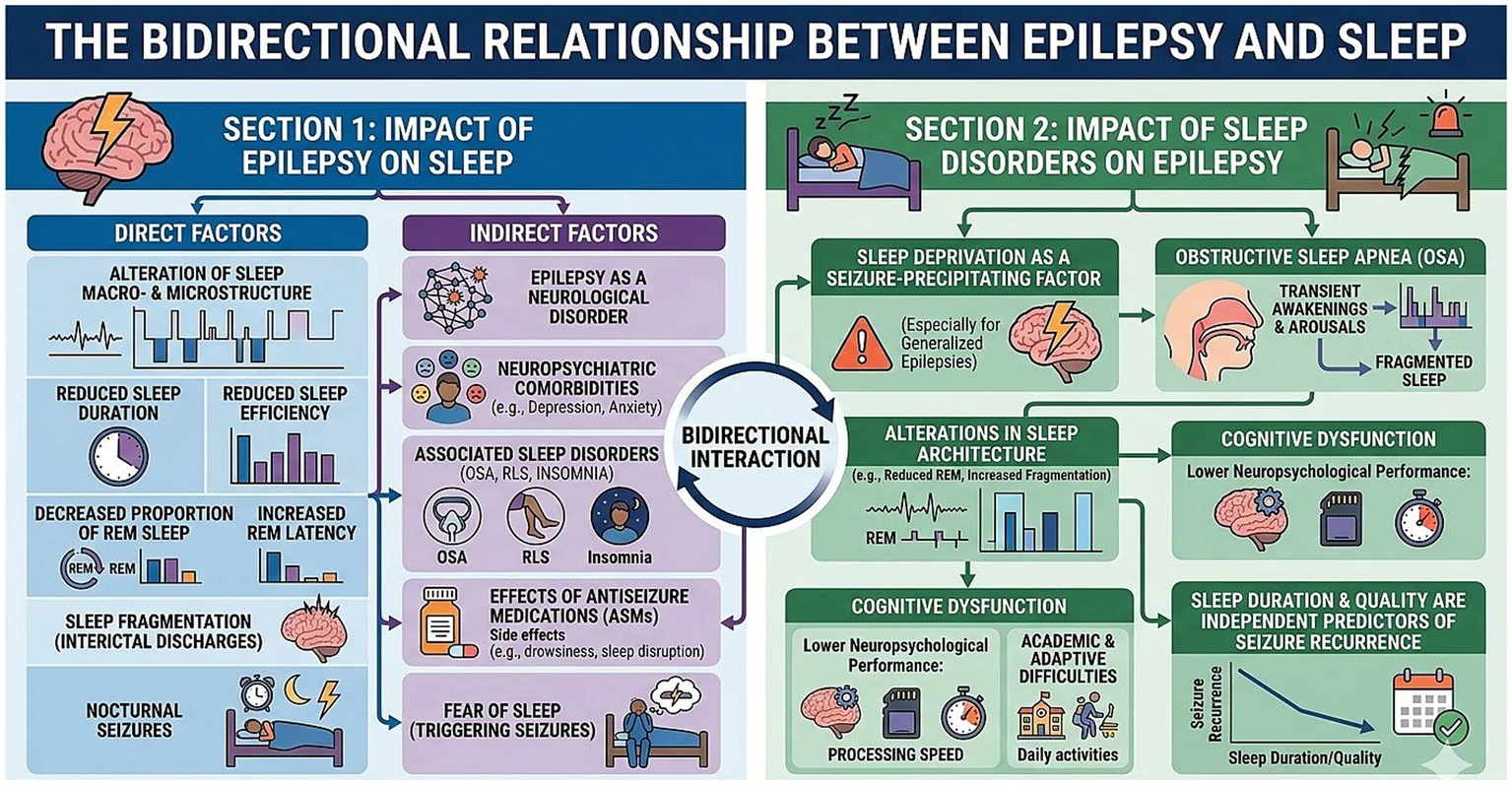
Schematic overview of the bidirectional relationship between epilepsy and sleep.

## 3 Epilepsy–sleep relationship

### 3.1 Sleep architecture and epileptiform activity

Sleep consists of a succession of cycles throughout the night during which changes in cerebral electrical activity manifest as alterations in consciousness, reduced responsiveness, and reduced muscle tone. It is divided into two main stages: REM (rapid eye movement) sleep and NREM (non-REM) sleep. REM sleep constitutes approximately 25% of total sleep time and is characterized by low-voltage activity, muscle atonia, and rapid eye movements, further divided into phasic REM (associated with vivid dreams) and tonic REM (a transitional state between phasic REM and wakefulness) (16). NREM sleep is divided into three stages. Stage N1 (≤5% of total sleep time) is characterized by low-voltage activity, theta activity, slow rolling eye movements, and vertex waves, representing the transition from wakefulness to sleep. Stage N2 (∼45% of total sleep time) is defined by sleep spindles (11–15 Hz) and K-complexes: spindles are indicative of synaptic plasticity and memory consolidation, while K-complexes serve to maintain sleep continuity and support memory consolidation. Stage N3, or slow-wave sleep (SWS, ∼25% of total sleep time), represents deep sleep and is characterized by high-voltage delta waves. Stages repeat in 4–6 cycles of approximately 90–120 min each (16, 17). Cerebral activity is characterized by neuronal network oscillations under both physiological and pathological conditions, classifiable as spikes and high-frequency oscillations (HFOs), further subdivided into ripples (80–200 Hz) and fast ripples (250–500 Hz). Under physiological conditions, HFOs are correlated with memory consolidation and sensorimotor processing. Continuous high-frequency activity in the background EEG reflects normal activity in regions such as the hippocampus and occipital lobe, while it is associated with epileptic activity when observed on a flat background. Interictal spikes derive from glutamatergic and GABAergic transmission, ripples are linked to GABAergic interneurons, and fast ripples reflect synchronous firing of excitatory neurons. Pathological HFOs coupling with interictal discharges reflect epileptic-type cortical excitability and may serve as biomarkers of epileptogenesis (18–20). Interictal epileptiform discharges are abnormal, transient, morphologically distinct waveforms generated by the summation of pyramidal cell potentials in a process known as the paroxysmal depolarization shift (PDS), characterized by synchronous changes in neuronal membrane potential. Their identification assists in characterizing the epilepsy type or syndrome (21). These elements represent a pathological variant of normal thalamocortical networks responsible for sleep microarchitecture organization (10).

### 3.2 Shared neurophysiological mechanisms

During NREM sleep, widespread cortical synchronization—particularly during stage N3—is necessary for generating physiological sleep graphoelements such as K-complexes and spindles, but it is also responsible for diffuse neuronal hyperexcitability that facilitates propagation of interictal epileptiform discharges, increasing both their amplitude and spatial spread. This sleep stage therefore promotes epileptic discharges and seizures by inducing thalamocortical oscillations to transform from sleep spindles into spike–wave discharges (10, 22, 23). The pro-epileptic role of NREM synchronization has been attributed to the low-frequency slow waves that represent the fundamental EEG graphoelement of NREM sleep. Epileptic events appear predominantly during the transition from the “up” state (associated with spindles and ripples) to the “down” state of slow waves. Large-amplitude slow waves have been shown to be specific modulators of epileptic activity: interictal spikes and HFOs are more frequent during high-amplitude diffuse slow waves than during low-amplitude ones, and in the epileptic brain, high neuronal synchronization episodes are associated with increased interictal spike and HFO frequency (24, 25). The hypothesis that spindles transform into spike–wave discharges has been challenged. Although both sleep spindles and spike–wave discharges originate from the cortico-thalamo-cortical circuit, their activation sites differ—thalamic for spindles and cortical for spike–wave discharges—and in most experimental models, spike–wave discharges evolve from EEG periods of increased theta band power, not from spindles (26). Notably, reduction in sleep spindle density has been shown to precede the onset of spike–wave discharges (27). Ictal and interictal activity are influenced differently by sleep: sleep exerts a substantially greater modulatory effect on interictal than on ictal activity, confirming that sleep does not influence epileptic activity uniformly (28). During REM sleep, by contrast, cortical desynchronization occurs through inhibition of thalamic synchronization, reducing neuronal excitability and limiting epileptiform discharge propagation. A review of 42 studies confirmed the protective role of REM sleep against seizure recurrence: focal seizures were 7.8 times more frequent during wakefulness, 87 times more frequent during N2, and 6.6 times more frequent during N3, compared to REM sleep (29).

### 3.3 Neurobiological basis of the epilepsy–sleep relationship

The neurobiological basis of the epilepsy–sleep relationship encompasses multiple levels of the nervous system, involving both neuroanatomical and functional dimensions: thalamocortical networks, circadian rhythm regulation systems, neurotransmitter and neuromodulator activity, synaptic plasticity, and the glymphatic system. Sleep spindles and K-complexes are two distinctive components of NREM sleep stage N2. K-complexes are generated predominantly in the frontal cortical regions and subsequently transmitted to the thalamus, where sleep spindles and delta waves are produced. Sleep spindles originate from GABAergic reticular neurons and are transmitted to the cortex via glutamatergic neurons, generating synchronous activity observable on EEG (30). The process of spindle generation involves three interacting components: thalamocortical neurons, reticulothalamic neurons, and pyramidal cells. During the transition from wakefulness to NREM sleep, reduced cholinergic transmission allows reticulothalamic neurons to enter burst-firing mode, generating inhibitory postsynaptic potentials in cortical neurons. This produces spindle oscillations that propagate to the cortex, amplified by corticothalamic feedback mechanisms (31). The GABAergic inhibition from reticular neurons can thereby induce cortical hypersynchronization in the form of spike–wave discharges (32). In the burst-firing mode of the thalamocortical system during NREM sleep, the reticular thalamic nucleus periodically inhibits relay thalamic nuclei, with the resulting sequence reflected at the cortical level as sleep spindles or delta waves. The further propagation of epileptiform discharges through intracortical circuits and their subsequent thalamic generalization demonstrates that thalamocortical networks not only synchronize physiological NREM activity but also predispose to seizure onset (23, 32). Notably, while thalamocortical dysfunction is classically associated with generalized absence epilepsy, these circuits may also be focally disrupted in focal epilepsy (33). At the molecular level, circadian rhythms are generated by a transcriptional–translational feedback circuit involving core “clock” genes present in almost all cells, which regulate the expression of ion channels and GABAergic receptors, influencing sleep structure and epileptic susceptibility (34). The expression of these genes, particularly *BMAL1*, *CLOCK*, *PER1*, *REV-ERBα*, and *RORα*, has been found to be associated with epilepsy, and mutations in circadian clock-related genes can cause syndromes that include epilepsy in their phenotype (35, 36). Clock genes coordinate neuronal excitability across the 24-h cycle by promoting in a temporally staggered manner the expression of ion channels responsible for depolarization and repolarization, thereby creating distinct windows of epileptic susceptibility within the sleep–wake cycle (31). The circadian and homeostatic regulation of seizure risk—and the role of the suprachiasmatic nucleus, cortisol, and melatonin in modulating neuronal excitability—are discussed in further detail in Section 5.1. The bidirectional relationship between sleep and epilepsy also involves numerous neuromodulators implicated in sleep–wake cycle regulation, including prostaglandin D2, melatonin, norepinephrine, serotonin, histamine, acetylcholine, orexin, and adenosine. Prostaglandin D2 is the most abundant prostaglandin in the brain, and its CSF levels increase in the context of sleep deprivation (30). Adenosine plays a critical role as an endogenous sleep neuromodulator and anticonvulsant, acting via two key receptor subtypes. A1 receptors, widely distributed throughout the brain, promote NREM sleep by inhibiting arousal and facilitating slow-wave sleep onset, and reduce epileptogenic potential by inhibiting excitatory transmission presynaptically and promoting membrane hyperpolarization postsynaptically (37, 38). A2A receptors, predominantly localized in the striatum, nucleus accumbens, and olfactory tubercle, stimulate sleep-promoting neurons in the ventrolateral preoptic area but may also promote epileptogenesis through membrane depolarization (39, 40). Adenosine levels correlate directly with time spent awake, serving as a homeostatic indicator of sleep pressure (41). Epileptic seizures increase adenosine release, and the resulting enhancement of adenosinergic tone attenuates neuronal hyperexcitability and reduces seizure propagation, underscoring the critical role of adenosine in controlling epileptic activity and the postictal refractory state (42, 43). Overexpression of adenosine kinase (ADK), which phosphorylates adenosine to AMP, limits A1 receptor activity and thereby stimulates neuronal excitability, suggesting a role in epileptogenesis; ADK inhibitors in the early stages of epileptogenesis have accordingly been proposed as potential antiepileptic agents (44). Orexin is an excitatory neuropeptide produced by the posterior lateral hypothalamic region that plays a fundamental role in sleep–wake regulation. Orexin levels are highest during wakefulness, reduced during slow-wave sleep, and absent during REM sleep, as orexinergic neurons suppress REM sleep; reduced orexin levels are a hallmark of type I narcolepsy (45). The loss of orexinergic tone permits the establishment of REM sleep, which appears to be protective against seizures as a phase of cortical desynchronization. Patients with epilepsy exhibit sleep–wake cycle dysregulation, sleep fragmentation, and insomnia, contributing to sleep instability and impaired REM sleep. A study in 40 patients with drug-resistant focal epilepsy demonstrated that baseline blood orexin levels were lower than in controls, while post-seizure levels—particularly after nocturnal seizures—were elevated, suggesting a close relationship with seizure occurrence (46). Given that seizure probability is higher during periods of elevated orexinergic tone and that REM sleep is associated with a lower seizure probability, orexin receptor antagonists have been proposed as novel therapeutic strategies against seizures, modulating both cortical excitability and sleep quality (47–49). NREM sleep is directly involved in neuronal plasticity and homeostasis. The synaptic homeostasis hypothesis (50) proposes that during NREM sleep, central nervous system synapses undergo downscaling of synaptic strength to permit associative plasticity during subsequent wakefulness. The progressive reduction of slow waves across the night reflects this process. When the mechanisms controlling neuronal hyperexcitability fail, homeostatic plasticity may become dysfunctional, and epileptogenesis therefore represents a distortion of NREM sleep–associated neuronal plasticity processes (22, 23, 32). The glymphatic system is a clearance system for interstitial fluids of the central nervous system, operating via paravascular pathways dependent on AQP4 channels on glial cells (51). In addition to eliminating neurotoxic waste accumulated during wakefulness, it facilitates the distribution of electrolytes, glucose, amino acids, and lipids (52). Glymphatic flow increases during NREM sleep, and certain AQP4 haplotypes are associated with changes in NREM sleep architecture (53, 54). Given that glymphatic activity depends on sleep and that sleep architecture is disrupted in epilepsy, patients with epilepsy may exhibit glymphatic dysfunction, which has been correlated with epileptogenesis through the accumulation of metabolic waste products (55). A study using DTI-ALPS (diffusion tensor image analysis along the perivascular space) demonstrated that glymphatic function was lower in patients with focal epilepsy than in healthy controls and was further reduced in those with poor response to antiseizure therapy, suggesting a link between glymphatic function and therapeutic efficacy (56). The glymphatic system may therefore offer new therapeutic horizons for both seizure control and sleep quality improvement.

## 4 Impact of epilepsy on sleep

It is well established that epilepsy has a direct impact on both the macro- and microstructure of sleep. In both focal and generalized epilepsies, epileptic discharges are associated with more unstable sleep, as a result of the direct effect of epilepsy itself, the underlying neurological condition, associated comorbidities, and the effects of ASMs (57, 58). Sleep fragmentation caused by interictal discharges disrupts the physiological role of sleep, impairing learning and cognitive function (59). Among the key markers of sleep instability is the Cyclic Alternating Pattern (CAP), a spontaneous and physiological NREM sleep rhythm characterized by electroencephalographic oscillations that contribute to sleep dynamics. CAP consists of transient arousal-related events alternating with intervals of background activity, and it is subdivided into phase A—comprising phasic sleep events—and phase B, corresponding to a return to background activity. Phase A is further classified into subtypes A1, A2, and A3, reflecting a progressive transition from synchronized to increasingly desynchronized EEG activity (60, 61). A meta-analysis of 11 clinical studies found an increase in CAP rates in patients with epilepsy. Specifically, the A1 subtype was lower in patients with both sleep-related and non-sleep-related epilepsy, while the A3 subtype was significantly more prevalent in patients with sleep-related epilepsy compared to healthy controls. The use of ASMs did not modify CAP microstates in patients with sleep-related epilepsy, and no significant differences were found in subtype proportions between treated and untreated patients (62). Over time, repeated seizures modulate the capacity of the cortex to participate in the physiological oscillations of sleep (63). Sleep efficiency is reduced in patients with both focal and generalized epilepsy. Patients with epilepsy show a decreased proportion of REM sleep, linked to epilepsy-induced alterations in thalamocortical systems, as well as overall impaired sleep quality. In addition, epileptic patients exhibit increased REM sleep latency and prolonged wake after sleep onset (WASO) (64, 65). At the clinical level, patients with epilepsy show reduced total sleep duration due to increased post-ictal nocturnal awakenings, a finding supported by evidence of heightened epileptic activity preceding arousals (12). Consequently, there is an increased risk of further seizure occurrence, since sleep deprivation represents one of the main seizure-triggering factors. Reduced sleep duration also manifests as excessive daytime sleepiness, with an inevitable impact on patients’ quality of life. Moreover, a higher prevalence of sleep disorders—including sleepwalking, parasomnias, and sleep-disordered breathing—has been reported in patients with epilepsy. In particular, there is a bidirectional relationship between epilepsy and obstructive sleep apnea (OSA), driven by the effect of epileptogenic discharges on respiration, the influence of ASMs on muscle tone, and the role of the vagus nerve in modulating upper airway muscle activity. In turn, sleep apnea causes sleep fragmentation and lowers the seizure threshold (66).

### 4.1 Effects of ASMs on sleep quality

Numerous studies have focused on the effects of ASMs on sleep architecture, with the dual aim of improving seizure control and sleep quality. ASMs can exert a wide range of effects on sleep—including drowsiness, particularly in the context of polytherapy, and in some cases insomnia—and may alter sleep architecture, especially REM sleep and slow-wave sleep, with a meaningful impact on cognitive function (67). In particular, calcium channel blockers and GABA enhancers appear to significantly increase slow-wave sleep compared to placebo; in addition, GABA enhancers are associated with a reduction in REM sleep, while calcium channel blockers tend to improve sleep efficiency. By contrast, sodium channel blockers, SV2A ligands, and broad-spectrum ASMs do not appear to substantially modify REM sleep, overall sleep architecture, or sleep efficiency (68). First-generation ASMs—including barbiturates, benzodiazepines, phenytoin, phenobarbital, carbamazepine, and valproate—reduce time spent in both REM sleep and slow-wave sleep, and fragment nocturnal sleep by increasing the number of arousals and transitions between sleep stages (69, 70). Both phenobarbital and phenytoin, for example, shorten sleep latency and reduce REM sleep. Newer-generation ASMs—including lamotrigine, levetiracetam, gabapentin, and topiramate—appear to have fewer effects on sleep architecture overall (71). Carbamazepine appears to exert transient adverse effects on sleep during the initial phase of treatment; however, after approximately 1 month of therapy, both sleep macrostructure and daytime alertness tend to normalize. This drug is reported to consolidate sleep through a reduction in arousals, an increase in slow-wave sleep, and a reduction in REM sleep (72, 73). Conversely, some studies in patients with temporal lobe epilepsy report that carbamazepine increases arousals and sleep instability through induction of CAP alterations (74). Lacosamide does not appear to significantly alter sleep architecture or daytime sleepiness; however, as monotherapy, it has been associated with a reduction in sleep latency and nocturnal awakenings, resulting in improved sleep quality (75, 76). Lamotrigine appears to increase REM sleep and reduce the transition to slow-wave sleep, without significant effects on daytime sleepiness, but has been associated with a higher risk of insomnia. Levetiracetam appears to have a dose-dependent effect on sleep, with improvements in sleep quality observed upon reaching the therapeutic target (72, 77). Cenobamate has a significant impact on sleep, primarily causing marked daytime sleepiness. The effect of valproate on sleep architecture remains controversial and appears to depend on epilepsy type, treatment duration, and therapeutic response; one study suggests that valproic acid has no significant effect on sleep per se (72). Polytherapy has been associated with poorer sleep quality, as it is linked to exacerbation of parasomnias and more severe sleep disturbances compared to monotherapy. In particular, patients with epilepsy using more than three ASMs show reduced sleep quality, which in turn adversely affects quality of life (78).

## 5 Impact of sleep disorders on epilepsy

Although numerous studies have analyzed the impact of epilepsy on sleep patterns, clinical and preclinical evidence on the short- and long-term effects of sleep disorders on epilepsy remains limited (79). In patients with epilepsy, sleep disorders are associated with a significant reduction in quality of life, independently of seizure frequency and type (7, 9, 11) contributing to an increased perception of disability and reduced participation in daily activities (80). In children with epilepsy, sleep impairment is additionally associated with worse caregiver-reported quality of life and a negative impact on psychological well-being and social and cognitive functioning (80). Alterations in sleep architecture—particularly reduced REM sleep and increased fragmentation—correlate with lower neuropsychological performance, especially in the domains of attention, memory, and processing speed, independently of seizure severity, contributing to academic and adaptive difficulties (81, 82). Persistent sleep disturbances over time are associated with more pronounced cognitive deficits and neurobehavioral multimorbidity, increasing the risk of developing affective symptoms such as anxiety and depression (81). Beyond the impact on quality of life, prospective studies have demonstrated that patients with epilepsy and poor sleep quality exhibit a higher seizure frequency and more rapid disease progression compared to those with adequate sleep (79, 83), and that sleep duration and quality are independent predictors of seizure recurrence (84).

### 5.1 Epilepsy and circadian rhythm

The circadian rhythm is an endogenous biological cycle of approximately 24 h that regulates numerous physiological and behavioral processes, including sleep, wakefulness, hormonal secretion, body temperature, metabolism, and cardiovascular function (34, 85, 86). At the molecular level these rhythms are generated by a network of clock genes present in almost all cells, which regulate ion channels and GABAergic receptors and thereby influence sleep structure and seizure susceptibility. At the systems level, the suprachiasmatic nuclei function as the central pacemaker, synchronizing endocrine rhythms via retinal light input (34, 87). Cortisol and melatonin are secreted according to circadian patterns and modulate neuronal excitability; cortisol concentrations are positively correlated with epileptiform activity in patients with stress-sensitive epilepsy and may contribute to the circadian pattern of seizure occurrence (34, 88). The suprachiasmatic nuclei also regulate sleep–wake propensity across the 24-h cycle, while sleep regulation additionally depends on the homeostatic process, which increases sleep pressure during wakefulness and reduces it during sleep. The interaction between these two opposing processes determines sleep structure and influences cortical excitability and seizure probability (34). Recent data suggest that an increased homeostatic sleep drive constitutes an independent factor capable of raising susceptibility to epileptic seizures (89). A modulatory role is played by the serotonergic system, particularly the 5-HT1A receptor expressed in sleep-promoting neurons. Under physiological conditions, activation of this receptor helps limit the increase in neuronal excitability associated with rising sleep pressure (90). Conversely, downregulation of 5-HT1A reduces this inhibitory control, rendering circuits more vulnerable and amplifying the proconvulsant effect of elevated homeostatic drive. Experimental evidence further indicates that pharmacological activation of the 5-HT1A receptor can attenuate this increase in risk (89). Long-term video-EEG monitoring over a 24-h period reveals incidence peaks that vary according to epilepsy type (generalized or focal), seizure-onset zone, and individual patient characteristics (34). In children and young adults (1–20 years), generalized seizures occur predominantly during wakefulness and daytime hours (91, 92). In mesial temporal lobe epilepsy, a bimodal distribution has been described with peaks in the morning and late afternoon, while frontal lobe seizures occur more frequently during NREM sleep (34, 93). Sleep–wake transitions are particularly critical: the shift from NREM sleep to wakefulness, or vice versa, is associated with increased cortical excitability and higher seizure probability, especially in frontal lobe epilepsy (34). As detailed in Section 3.2, interictal epileptiform discharges intensify during slow-wave NREM sleep (N3) due to high thalamocortical synchronization, while REM sleep exerts a relatively protective effect against epileptic activity (15). NREM sleep may facilitate seizures particularly in genetic or lesional forms of epilepsy, independently of the seizure-onset zone (94). Limited data are available on the circadian distribution of occipital and parietal lobe seizures; however, occipital seizures appear to occur preferentially during the day and during wakefulness (91, 92). The influence of the seizure-onset zone on timing was also demonstrated in a case study of a 57-year-old woman with focal seizures from two independent foci, one limbic and one non-limbic, each characterized by a distinct circadian pattern (95). However, these studies do not allow definitive conclusions regarding whether seizure timing is modulated by behavioral states or by the circadian rhythm itself, and resolving this question would require complex experimental protocols (34).

### 5.2 Sleep deprivation as a seizure-precipitating factor

Sleep deprivation (SD) is universally regarded as a factor capable of increasing seizure frequency (15) and is widely used as an EEG activation procedure for diagnostic purposes (79). In a prospective case–control study of 144 patients admitted for epileptic seizures, sleep duration in the 24 h preceding the event was significantly shorter (7.3 h) compared to seizure-free periods (8.3 h; *p* < 0.0005), and SD was confirmed as an independent precipitating factor even after adjustment for alcohol consumption and antiseizure medication use (96). Similarly, a prospective study of 209 patients with newly diagnosed epilepsy found that short sleep duration at baseline was associated with more than a doubled risk of seizure recurrence, following a U-shaped relationship with minimum risk around 8 h of sleep per day (84). However, the relationship between SD and seizure risk is not uniform: in some cohorts, only a minority of patients show a significant correlation between sleep duration and seizure risk, while variations in sleep onset and wake times appear to be of greater relevance (97). The impact of SD appears particularly marked in temporal lobe epilepsy, where seizure risk may increase by up to 100% on days of deprivation (98). From a pathophysiological standpoint, prolonged wakefulness increases cortical excitability, particularly in patients with epilepsy, with syndrome-specific effects (99). One of the mechanisms involved concerns the reduction of tonic GABAergic inhibition (TGI) in dentate circuits (83). TGI, regulated by GABAA receptors containing α4/δ subunits in the dentate gyrus and α5/γ subunits in CA1, contributes more substantially to overall inhibition than the phasic component; SD reduces the expression of α5 and δ subunits, diminishing TGI and increasing seizure susceptibility (83). Concurrently, SD leads to an increase in proinflammatory cytokines (TNF-α, IL-1β, IL-6) and oxidative stress, which lower the seizure threshold by promoting increased glutamate release and reduced GABAergic inhibition (100, 101). SD may thus act as a further inflammatory “second hit,” contributing to a vicious cycle of neuroinflammation, neurodegeneration, and increased epileptic susceptibility (79). Finally, dysfunction of the glymphatic system in the context of SD may promote the accumulation of pro-epileptogenic substances and increase seizure risk (32).

### 5.3 Obstructive sleep apnea and seizure control

Obstructive sleep apnea (OSA) is prevalent in patients with epilepsy, at rates exceeding those in the general population (101). Episodes of OSA can produce intermittent periods of hypoxia and hypercapnia that induce transient awakenings and arousals, causing sleep fragmentation and consequent SD, with lowering of the threshold for epileptic seizures (102). At the termination of each apnea event, arousal provokes a peak in sympathetic activation and a reduction in vagal activity, resulting in fluctuations in heart rate and blood pressure. According to the American Heart Association, these autonomic events are involved in the pathogenesis of cardiovascular complications and may increase seizure susceptibility (103). The use of continuous positive airway pressure (CPAP) ventilation is associated with a significant reduction in seizure frequency and improved seizure control, both in patients with controlled and refractory epilepsy (101). Screening for OSA in patients with epilepsy is strongly recommended, particularly in the presence of risk factors such as obesity, advanced age, diabetes, or a history of excessive daytime sleepiness (79).

## 6 Specific clinical conditions: principal epileptic syndromes associated with sleep

Approximately 10–15% of epilepsies are sleep-related. These syndromes share a close relationship between sleep and epileptic activity, with EEG patterns and clinical manifestations that vary according to sleep stage and epilepsy type (104, 105).

These specific conditions can be subdivided into three categories: sleep-related epilepsies, sleep-accentuated epilepsies, and awakening-related epilepsies (11).

The principal sleep-related epileptic syndromes include the following:

- Childhood epilepsy with centrotemporal spikes (SeLECTS), formerly known as Rolandic epilepsy: seizures, typically focal motor or sensory, occur predominantly during NREM sleep and interictal epileptic activity is accentuated during slow-wave sleep.
- Self-limited epilepsy with autonomic seizures (SeLEAS), formerly known as Panayiotopoulos syndrome: characterized by autonomic seizures, often accompanied by vomiting and altered consciousness, manifesting chiefly during sleep, with accentuation of EEG anomalies during slow-wave sleep (106).
- Sleep-Related Hypermotor Epilepsy (SHE).

The principal sleep-accentuated epileptic syndromes include the following:

- Lennox–Gastaut syndrome: nocturnal tonic seizures are a defining feature, with accentuation of EEG anomalies during sleep.
- Landau–Kleffner Syndrome (LKS).
- Epileptic Encephalopathy with Spike–Wave Activation in Sleep (EE-SWAS/CSWS/ESES).
- Infantile epileptic spasms syndrome spasms occur frequently on awakening or during sleep, and the characteristic hypsarrhythmia on EEG may be visible only during sleep.

The third group comprises awakening-related epilepsies, and includes the following:

- Juvenile myoclonic epilepsy: myoclonic and tonic–clonic seizures typically occur in the first hours after awakening, frequently precipitated by sleep deprivation.
- Epilepsy with generalized tonic–clonic (GTC) seizures alone, in which most seizures occur shortly after awakening from sleep.

The following sections analyze in detail certain forms of nocturnal epilepsy that represent the principal sleep-related epilepsies of clinical relevance, with significant impact on neurocognitive and behavioral development. Active forms of these epilepsies are almost exclusively pediatric in onset; in adults, one observes primarily residual neuropsychological sequelae, without new clinical manifestations typical of the active syndromes.

### 6.1 Sleep-related hypermotor epilepsy

Previously known as nocturnal frontal lobe epilepsy (NFLE), SHE was renamed in 2016 following an international consensus conference, after it became clear that approximately 30% of cases have an extrafrontal origin and that seizures are linked to sleep per se rather than to a circadian pattern of occurrence (107). The renaming also reflected the characteristic semiology, dominated by hypermotor seizures rather than merely frontal lobe involvement. At the present state of knowledge, SHE is best regarded as a single syndrome, defined by its clinical manifestations—hypermotor seizures occurring predominantly during sleep—and possibly resulting from shared downstream mechanisms involving sleep–wake oscillation changes, representing a distinct pathogenic network (94). SHE is a focal epilepsy with onset typically in the first two decades of life (peak at ages 11–14 years, range from 2 months to 64 years), with a slight male predominance. Seizures occur predominantly during NREM sleep, especially stage N2 (108). Neurological examination and cognitive function are typically preserved, though psychiatric comorbidities and cognitive difficulties may be present in a subset of patients. Clinical manifestations encompass three principal subtypes: paroxysmal arousals (brief episodes of sudden motor arousal), nocturnal paroxysmal dystonia (attacks with complex dystonic-dyskinetic features), and episodic nocturnal wanderings (stereotyped, agitated sleepwalking-like behavior) (109). Seizures may last from a few seconds to approximately two minutes, often occurring in clusters during a single night, with prominent autonomic activation. A range of additional ictal features may be observed, including vigorous proximal limb and trunk movements, facial grimacing, emotional expressions, vocalizations, and, in some cases, retained awareness during ictal events (110). An important clinical clue distinguishing SHE from NREM parasomnias is the stereotyped, brief, and often clustered nature of events: NREM parasomnias, by contrast, tend to be prolonged (often exceeding 30 min), non-stereotyped, and typically occur only once per night. The extrafrontal epileptogenic zone documented in multiple series includes the temporal and parietal lobes and the insula, underscoring the challenge of localizing the seizure-onset zone on clinical grounds alone. SHE encompasses both genetic and structural etiologies. Structural causes, particularly focal cortical dysplasia (FCD), account for approximately 16.5% of cases. Autosomal dominant familial forms (ADSHE) have been identified, associated with pathogenic variants in genes encoding ion channel subunits, notably *CHRNA4*, *CHRNB2*, and *KCNT1*, reflecting the critical role of neuronal nicotinic acetylcholine receptor (nAChR) subunits in the pathophysiology of this condition (111). Additional genetic causes, including variants in *DEPDC5* (associated with mTOR pathway dysregulation), have been described in both familial and sporadic cases. Diagnosis rests primarily on video-PSG or video-EEG during sleep. Brain MRI is recommended in all patients to exclude structural lesions, even if normal in the majority of cases. Genetic testing should be considered, particularly in familial presentations. First-line pharmacological treatment is carbamazepine, which achieves complete seizure remission in approximately 20% of cases and significant reduction (>50%) in a further 48% (108). In patients with an unsatisfactory response to monotherapy, topiramate and lacosamide represent reasonable add-on strategies. Oxcarbazepine and eslicarbazepine acetate are considered comparable alternatives given their similar mechanism of action. Approximately 30% of patients develop drug-resistant epilepsy. In carefully selected drug-resistant cases with an identifiable epileptogenic zone, particularly FCD, epilepsy surgery offers the prospect of significant improvement, with up to two-thirds of operated patients achieving meaningful seizure reduction or freedom (110).

### 6.2 Epileptic encephalopathy with spike–wave activation in sleep (EE-SWAS/CSWS/ESES)

The ILAE Task Force on Nosology and Definitions has recently proposed replacing the terms ESES (Electrical Status Epilepticus during Sleep) and CSWS (Continuous Spike-and-Waves during Sleep) with the umbrella designation “epileptic encephalopathy with spike-wave activation in sleep” (EE-SWAS) (112). The term DEE-SWAS (developmental and epileptic encephalopathy with spike–wave activation in sleep) is used when there is pre-existing neurodevelopmental impairment prior to regression, distinguishing it from EE-SWAS, which applies when prior development was normal. This nosological revision removes the connotation of “status epilepticus” from descriptions of an EEG phenotype whose precise clinical significance varies according to context. EE-SWAS is characterized by cognitive, behavioral, and motor deterioration in conjunction with marked activation of epileptic EEG anomalies during NREM sleep (113). This syndrome has a peak age of onset between 4 and 5 years, affects both sexes, and the EEG pattern as well as seizures typically abate around puberty; however, associated cognitive and behavioral deficits frequently persist. The condition encompasses a broad phenotypic spectrum: all cognitive domains may be affected, including language and communication, temporal–spatial orientation, attention, and social interaction. Some affected children do not experience electroclinical seizures, with the encephalopathy driven primarily by the epileptiform EEG burden during sleep. According to the ILAE position paper (112), EE-SWAS is defined by a substantial increase in epileptiform discharges in drowsiness and non-REM sleep, presenting as slow spike-and-wave complexes at a frequency of 1.5–2 Hz, consistently observed also during Stage II sleep. Nowadays the original EEG criterion of a spike wave index (SWI) > 85% of slow wave sleep is no longer considered valid. The distribution of this abnormal activity is predominantly diffuse, although focal (particularly frontal) or multifocal patterns may occasionally be encountered. During REM sleep, the epileptiform discharges tend to decrease considerably or may disappear. The physiological hallmarks of normal sleep architecture are either absent or no longer clearly identifiable. Neuropsychological impairment is frequently irreversible if not treated promptly. A shorter duration of active EE-SWAS prior to treatment initiation is associated with better cognitive outcomes (114). Landau–Kleffner syndrome (LKS) is currently classified as a distinct variant within the EE-SWAS spectrum, characterized by a deterioration that primarily involves language functions through the development of an acquired auditory agnosia (112). A central pathophysiological hypothesis holds that the cognitive deterioration observed in EE-SWAS results from spike-induced disruption of the synaptic homeostasis processes that normally occur during slow-wave sleep. According to the synaptic homeostasis hypothesis (115) continuous epileptiform activity during NREM sleep interferes with synaptic downscaling, an interference particularly deleterious during the critical developmental period. The reversibility of cognitive deficits following resolution of the EEG pattern—observed in longitudinal studies—provides indirect support for this hypothesis. Therapeutic options include ASMs, corticosteroids, the ketogenic diet, and, rarely, surgery (116). Among treated patients, those most likely to benefit from corticosteroids are children with acute or subacute cognitive regression, normal development prior to onset, and absence of structural brain abnormalities. A multicenter European randomized controlled trial (RESCUE ESES) demonstrated that corticosteroids are superior to clobazam in improving cognitive function as initial therapy (117, 118). Other therapeutic options include high-dose benzodiazepines, valproate, and levetiracetam. Treatment choices must balance efficacy in reducing epileptiform activity against potential adverse effects. The ketogenic diet is particularly useful in children with corticosteroid-dependent or corticosteroid-resistant EE-SWAS, allowing in some cases steroid withdrawal while maintaining or improving outcomes (119). Immunotherapy with intravenous immunoglobulins may be considered in children with EE-SWAS refractory to conventional therapies, particularly in those with *GRIN2A* variants or suspected autoimmune pathogenesis; evidence remains limited and response is variable (120).

## 7 Parasomnias vs. nocturnal epileptic seizures: differential diagnosis

Parasomnias are sleep disorders characterized by undesirable physical or behavioral events occurring during sleep, in specific stages, or during sleep–wake transitions, and not under voluntary control (121). They are traditionally classified according to the sleep stage of origin. NREM parasomnias, or arousal disorders, include confusional arousals, sleepwalking, sleep terrors, and sleep-related eating disorder (121, 122). These events arise from incomplete cortical awakening during deep NREM sleep, often in the presence of factors promoting sleep fragmentation such as stress, OSA, or environmental noise (15). REM parasomnias include REM sleep behavior disorder (RBD), recurrent isolated sleep paralysis, nightmare disorder, and sleep-related hallucinations (121, 122). Among these, RBD is most clinically relevant and is characterized by loss of REM atonia due to dysfunction of pontine REM-promoting neurons, leading to dream enactment. In contrast, recurrent isolated sleep paralysis reflects persistence of REM atonia during wakefulness (122). Parasomnias are generally more frequent in children, except for RBD, which is more common in males over 50, whereas nocturnal epilepsies typically begin in adolescence or early adulthood (123). The differential diagnosis between parasomnias and sleep-related epilepsies is challenging due to overlapping clinical manifestations such as sudden arousals, confusional states, complex movements, vocalizations, and ambulation (15, 124). RBD may closely mimic ictal events because of complex motor and vocal behaviors. NREM parasomnias and epileptic seizures are often associated with amnesia, making witness reports crucial and history-taking more difficult (15). Coexistence of the two conditions in the same individual further complicates diagnosis (125). Indeed, recent evidence suggests a pathophysiological continuum between parasomnias and SHE, as both are associated with sleep instability, slow-wave fragmentation, altered NREM–REM balance, and shared genetic predisposition (e.g., *CHRNA4*). A thorough clinical history is the first diagnostic step and may be sufficient in typical cases. Otherwise, diagnosis requires integration of clinical data with video-PSG with EEG montage, which represents the gold standard for the differential diagnosis (123, 126). A single recording may be insufficient to capture events or EEG abnormalities (123). Home video recordings are therefore useful, especially in patients with infrequent episodes, and should document at least one or two major events (15, 123). More detailed information of differential diagnosis between Disorders of Arousal (DoA) and SHE is shown in Table 1.

**Table 1:** Differential diagnosis between disorders of arousal and sleep-related hypermotor epilepsy (15, 126, 128).

Feature	Disorders of arousal (DOA)	Sleep-related hypermotor epilepsy (SHE)
Clinical features		
Age at onset	Childhood, usually <10 years	Adolescence or adulthood
Sex	Both	Male predominance
Positive family history of NREM parasomnias	62–96%	39%
Etiology	Age-related maturational disorder of NREM sleep	Cortical focal dysplasia or genetic epilepsy
Frequency of events	Typically once per night; <1–4 per month	Highly variable; may cluster or recur multiple times per night; ~20–40 per month
Daytime consequences	Excessive daytime sleepiness, mood disorders, migraine, physical injuries	Excessive daytime sleepiness, mood disorders, physical injuries
Nocturnal events		
Timing in the night	First third of the night (usually after 90 min of sleep)	No consistent distribution; may occur within first 30–60 min
Trigger factors	External stimuli (noise), sleep deprivation, febrile illness, alcohol, stress, OSA	Often none
Onset	Gradual arousal—eye opening, head lifting, stretching, yawning	Sudden, abrupt paroxysmal arousal (median ~5 s duration)
Motor pattern	Non-stereotyped; ranges from simple arousal movements (SAMs) to complex ambulatory behaviors (CAMs); motor arrest common	Stereotyped, often violent; tonic-dystonic posturing or hypermotor pattern; high inter-episode similarity
Episode duration	Seconds to 30 min	Seconds to minutes (often <2 min; SPAs median ~5 s)
Interaction with environment	Possible response to external stimulation or verbal commands; ability to navigate familiar environment	Poor and incidental
Spontaneous awakening after event	Infrequent	Possible
Consciousness if awakened	Impaired	Normal
Recall of event	None (amnesia typical; witness accounts essential)	Variable; postictal confusion common
Post-ictal signs	Absent	May include incontinence, Todd’s paralysis, language disturbance
Video-polysomnographic features (126)		
Sleep stage at onset	Predominantly NREM N3 (slow-wave sleep)	Predominantly NREM N1–N2 (light sleep)
Number of motor events/night	Lower: mean 3.2 ± 2.4 (total events per recording)	Higher: mean 6.9 ± 8.3 (total events per recording)
Major events outside N3	Very rare (mean 0.05 ± 0.22); highly specific for SHE if present	Common (mean 3.10 ± 4.89); ≥1 major event outside N3
Minor events during N3	≥1 minor event during N3 strongly suggestive for DOA (mean 1.81 ± 1.94)	Minor events during N3 infrequent (mean 0.53 ± 1.25)
Timing distribution (Events Distribution Index)	Events tend to cluster in the first half of the night (index 0.50 ± 0.66)	Events more evenly distributed or later in the night (index 0.14 ± 0.71)
N3 Distribution Index	N3 predominantly in first part of night (index 0.38 ± 0.24)	N3 less concentrated in first part of night (index 0.17 ± 0.37)
EMG/autonomic activation	Motor activity on EMG; limited autonomic activation	Motor activity on EMG + autonomic activation (tachycardia, polypnea, vasoconstriction on plethysmography)
EEG findings		
Interictal EEG	Epileptiform discharges occasionally present (not diagnostic); ictal slow waves	Interictal abnormalities absent in up to 51%; ictal EEG often normal or obscured by movement artifacts; frankly epileptiform ictal rhythms in <50%
Diagnosis and prognosis		
Diagnostic approach	Clinical history + video-PSG; ≥1 minor event during N3 strongly suggestive	Integration of clinical, video-PSG, and EEG data; ≥1 major event outside N3 most accurate single predictor
Prognosis	Tends to resolve by adolescence	Often stable or persistent with increasing age

DOA, disorders of arousal; SHE, sleep-related hypermotor epilepsy; SAMs, simple arousal movements; CAMs, complex ambulatory movements; SPAs, sleep-related paroxysmal arousals; PSG, polysomnography; OSA, obstructive sleep apnea; N1/N2/N3, NREM sleep stages.

## 8 Diagnostic tools for the study of sleep in epilepsy and sleep disorders

### 8.1 Polysomnography and prolonged EEG

PSG combined with an expanded EEG montage is a cornerstone investigation for evaluating the bidirectional relationship between sleep and epilepsy, enabling simultaneous assessment of sleep architecture and epileptiform activity (64). An International 10–20 montage with ≥18 channels significantly improves diagnostic yield (127). The American Academy of Sleep Medicine recommends PSG with expanded EEG in children when clinical evaluation and routine EEG are inconclusive (128). Combined video-EEG-PSG studies show abnormalities in up to 94% of epilepsy patients with sleep complaints, with EEG abnormalities in 53% and seizures recorded in 40% (129). Objective PSG findings consistently show altered sleep macrostructure, including reduced total sleep time, lower efficiency, increased fragmentation, and redistribution of sleep stages (64). Focal epilepsy is associated with reduced REM sleep and efficiency, whereas generalized epilepsy shows increased slow-wave sleep and reduced efficiency (130). Drug-resistant epilepsy presents more severe disruption, with reduced N3 and REM sleep and increased wake after sleep onset, especially in cognitively impaired patients (131). Microstructural analysis using CAP demonstrates increased NREM instability, with higher CAP rates and reduced A1 subtypes (62). Sleep-related epilepsy shows increased A3 subtypes, while ASMs may partially mitigate NREM instability. Prolonged EEG confirms that interictal discharges peak during sleep regardless of location, whereas seizure timing varies by onset zone (34, 132). Comorbid sleep disorders are common: sleep-disordered breathing occurs in 27–80% of children and obstructive sleep apnea in 21–22% of adults undergoing PSG-EEG (128, 129, 133). Their recognition is of direct clinical relevance, as they may independently worsen seizure control, cognition, and quality of life.

### 8.2 Actigraphy

Actigraphy, based on wrist accelerometry, provides a non-invasive method for assessing sleep–wake patterns over extended periods in ecologically valid settings, complementing laboratory-based PSG. In epilepsy, it quantifies rest-activity rhythms, circadian organization, and sleep duration over days to weeks. Validation studies in children show strong correlations with video-EEG for key variables, supporting its utility as a reliable objective clinical tool for sleep evaluation in this population (134). In adults with focal epilepsy, actigraphy reveals reduced rhythm amplitude and increased variability, associated with seizure intractability and polytherapy (134). In drug-resistant epilepsy, increased wake after sleep onset correlates with higher seizure frequency, longer disease duration, depressive symptoms, and poorer quality of life. Improvements in sleep continuity after therapy adjustments are associated with reduced seizure frequency, supporting a bidirectional relationship. An important technical caveat concerns the limited specificity of actigraphy for detecting wakefulness after sleep onset: ictal and peri-ictal motor activity may be misclassified by standard scoring algorithms, particularly in patients with frequent nocturnal seizures. Actigraphy-derived sleep efficiency estimates should therefore be interpreted with caution in this population and, where possible, cross-validated with video or EEG data. Emerging wearable devices integrating accelerometry with physiological sensors (e.g., PPG, temperature, electrodermal activity) improve sleep staging and may enable non-invasive seizure detection. Some FDA-cleared devices are already available, and early data suggest that reduced sleep efficiency may predict seizure occurrence in some patients.

### 8.3 Validated questionnaires for sleep disorders

Validated questionnaires are widely used for screening sleep disturbances in large cohorts of neurological patients owing to their low cost, ease of administration, and ability to capture the subjective experience of sleep quality (135). Their systematic use in epilepsy monitoring units identifies previously unrecognized sleep and psychiatric comorbidities and influences management in over 50% of cases (136). The Pittsburgh Sleep Quality Index (PSQI) is the most widely used tool. A meta-analysis encompassing 25 studies and 8,196 individuals demonstrated significantly higher PSQI scores in patients with epilepsy (pooled mean difference 1.27), approaching the established pathological threshold of 5 (137). Higher PSQI scores correlate with seizure frequency, medication burden, anxiety, depression, and reduced quality of life. Certain drugs (e.g., lamotrigine, phenobarbital) and polytherapy are associated with worse sleep quality (78). The Epworth Sleepiness Scale (ESS) demonstrates satisfactory internal consistency (Cronbach’s α = 0.75) (138). Although overall ESS scores do not differ significantly from those of controls (137), subgroup analyses show higher scores in generalized epilepsy and sex-specific associations with medication dose and OSA risk (139). Excessive daytime sleepiness (ESS > 10) occurs in 20–25% of patients (140). Additional tools include the Insomnia Severity Index (ISI), which demonstrates significantly higher insomnia prevalence in epilepsy patients than controls (OR 5.91) and a correlation with seizure activity (141), as well as the Groningen Sleep Quality Scale, MOS-Sleep, and Sleep Diagnosis List, all indicating higher rates of sleep impairment in epilepsy (7). Several important methodological limitations characterize the current evidence base on subjective sleep assessment in epilepsy. These include heterogeneity of instruments and patient populations, limiting cross-study comparability (7, 135, 137); subjective bias influenced by psychological factors (135, 141, 142); lack of cross-validation against objective measures such as PSG or actigraphy (64, 143); and inconsistent reporting practices regarding cut-offs, definitions, and confounding variables (135, 144, 145).

The complementarity between subjective questionnaire-based and objective instrumental assessments is increasingly recognized in the literature and in clinical guidelines. The EAN, ESRS, and ILAE-Europe have jointly proposed a stepwise diagnostic pathway recommending systematic questionnaire-based screening (PSQI, ESS, ISI) as the initial assessment in all patients with epilepsy and sleep complaints, followed by objective evaluation—PSG with expanded EEG montage, video-EEG, or actigraphy—in cases where clinical suspicion is not resolved by the initial screen, or where epilepsy is refractory or associated with neuropsychiatric comorbidity (146). However, these recommendations are based on expert consensus rather than randomized evidence, and no standardized framework exists.

## 9 Sleep EEG biomarkers: prognostic and therapeutic implications

An evolving area of clinical relevance concerns the identification of EEG biomarkers of epilepsy evolution and surgical outcome detectable through polysomnography or prolonged EEG recording. Among the most promising candidates are spike–wave frequency, delta band power, sleep spindle reduction, increased gamma band power, and the H (Hurst) exponent—a non-oscillatory, scale-free measure of background EEG complexity. In a study of 38 adults with drug-resistant epilepsy undergoing intracranial EEG with a subset proceeding to resective surgery, distinct oscillatory and non-oscillatory sleep EEG metrics were found to behave differently across the epileptic network, with the H exponent emerging as one of the most accurate biomarkers for classifying epileptogenic areas resected in patients with good postsurgical outcome (147). These findings suggest that the absence or attenuation of normal sleep oscillatory physiology may provide information complementary to pathological epileptic markers in delineating the epileptogenic zone. In children, automated quantification of spike–wave activity during sleep has been proposed as a tool for predicting progression towards EE-SWAS years before formal clinical diagnosis, potentially enabling earlier therapeutic intervention (148). The relationship between sleep spindle density and cognitive outcome is also gaining traction as a clinically informative biomarker, particularly in infantile epileptic spasms syndrome, where persistent absence of sleep spindles on sequential EEG recordings is associated with poor neurodevelopmental outcomes, while their re-emergence following effective treatment correlates with improved cognitive trajectories. In adults, reduced sleep spindle density and increased gamma power correlate with the extent of the epileptic network; the H exponent demonstrates among the highest accuracy for identification of epileptogenic zones subsequently resected with favorable outcome (147). High-frequency oscillations (HFOs), particularly spike-coupled ripples recorded during interictal sleep, represent an additional biomarker class of increasing relevance to presurgical evaluation, with growing evidence that resection of HFO-generating tissue is associated with superior postsurgical seizure freedom, although the optimal clinical integration of HFO analysis remains an active area of methodological development. Further sleep EEG biomarkers of clinical interest include alterations in REM latency associated with short-term seizure risk, and respiratory and cardiac autonomic features during sleep as potential indicators of sudden unexpected death in epilepsy (SUDEP) risk (149–151). While these markers hold considerable promises for prognostic stratification and longitudinal monitoring, their clinical translation requires further prospective validation in adequately powered cohorts before they can be incorporated into routine clinical practice.

## 10 Therapeutic management

The therapeutic management of epilepsy in relation to sleep requires a personalized approach encompassing optimization of ASM therapy, active treatment of comorbid sleep disorders, adoption of behavioral and sleep hygiene interventions, and—in selected patients—the adjunctive use of melatonin and other chronobiological strategies.

### 10.1 Optimization of antiseizure medication therapy in relation to sleep

Current guidelines recommend that the dose and administration timing of ASMs be optimized with the dual objective of improving both sleep quality and seizure control (146). Personalization of pharmacological management is particularly important in patients with predominantly nocturnal epilepsy, in whom chronotherapeutic strategies—such as a weighted evening dose—may confer additive benefits on nocturnal seizure control, as has been demonstrated for valproate (72, 77, 152).

#### 10.1.1 Effects of specific ASMs on sleep

The effects of individual ASMs on sleep architecture represent an important dimension of treatment selection, particularly when sleep quality is already compromised. Adverse sleep-related effects are typically dose-dependent and more frequent with polytherapy regimens (78, 152). Reported adverse effects include insomnia, excessive daytime sleepiness, sleep fragmentation, alterations in REM and NREM sleep distribution, and cognitive disturbances (68, 70, 72, 77, 153). Phenobarbital, primidone, benzodiazepines, and phenytoin are associated with marked sedation and sleep fragmentation, particularly at higher doses and in elderly patients (68, 152). Lamotrigine carries a recognized risk of insomnia and sleep-onset difficulties, attributed to its activating pharmacological profile, and this effect is more prominent at higher doses or when taken in the evening (77, 78, 152, 153). Phenytoin and valproate may reduce N3 and REM sleep (154). Carbamazepine and oxcarbazepine are generally considered neutral or minimally disruptive to sleep architecture (78, 152). In contrast, gabapentin, tiagabine, pregabalin, and clobazam tend to improve sleep efficiency and increase slow-wave sleep, a profile that may be advantageous in patients with comorbid insomnia or sleep fragmentation (72, 77, 152). Eslicarbazepine acetate, lacosamide, and perampanel appear to have a neutral or mildly beneficial effect on sleep (154). Levetiracetam does not significantly alter slow-wave or REM sleep proportions (72). Polytherapy is independently associated with a higher prevalence of both insomnia and excessive daytime sleepiness (78, 155).

#### 10.1.2 Interactions between ASMs and non-pharmacological treatments

Meaningful interactions exist between ASMs and non-pharmacological treatment strategies that may further modulate sleep quality (70, 72, 77, 154). Epilepsy surgery and the ketogenic diet tend to improve sleep architecture following successful intervention, while vagus nerve stimulation (VNS) may paradoxically increase the risk of sleep-disordered breathing, particularly in patients with pre-existing upper airway vulnerability, through direct stimulation of the recurrent laryngeal nerve and consequent intermittent pharyngeal obstruction during stimulation cycles (70, 78). Clinicians implanting or managing VNS should therefore include prospective sleep evaluation—including home sleep testing—as part of the routine follow-up protocol.

### 10.2 Treatment of comorbid obstructive sleep apnea

The treatment of comorbid OSA in patients with epilepsy represents one of the most clinically impactful interventions available in this setting. CPAP therapy is associated with a meaningful reduction in seizure frequency and improvement in sleep quality. Retrospective and prospective series consistently demonstrate that CPAP-adherent patients achieve superior seizure control (78, 155, 156). OSA promotes intermittent hypoxemia, cortical arousal instability, and REM sleep fragmentation—all of which lower seizure threshold—and CPAP reversal of these processes reduces the epileptogenic burden conferred by sleep disruption. Early identification and treatment of OSA is therefore strongly recommended, particularly in those with drug-resistant diseases (157). In patients who cannot tolerate CPAP, mandibular advancement devices represent an alternative, though evidence in epilepsy-specific populations remains limited. VNS may precipitate or worsen OSA by producing intermittent upper airway obstruction synchronous with the stimulation cycle (157).

### 10.3 Sleep hygiene and behavioral interventions

Sleep hygiene measures and behavioral interventions—including stabilization of sleep–wake schedules, reduction of sleep deprivation, and stress management—are recommended for all patients with epilepsy as a component of comprehensive care. Randomized trials demonstrate improvements in both sleep efficiency and total sleep time in children with epilepsy following structured behavioral-educational sleep programs (3, 146, 158). European scientific societies emphasize the importance of systematic evaluation of sleep habits as part of the routine epilepsy consultation (77). Behavioral strategies alone, however, have recognized limitations in many patients with epilepsy. Sleep quality is frequently compromised by nocturnal seizures, interictal epileptiform activity, and the adverse effects of ASMs—factors not amenable to behavioral modification alone (3, 135, 146, 159). Individual variability in sleep architecture and seizure patterns further necessitates personalized adaptations of behavioral protocols (97). The co-occurrence of sleep disorders—including OSA, parasomnias, and insomnia—requires a more complex, multimodal approach, in which sleep hygiene serves as a foundation but must be integrated with targeted diagnostic evaluation and treatment of the underlying sleep disorder (135, 146, 159). Certain patient groups present greater challenges for adherence to and effectiveness of sleep hygiene interventions. Patients with drug-resistant epilepsy show marked sleep fragmentation and NREM microstructural instability that substantially reduce the efficacy of behavioral approaches alone (64). Those with comorbid neuropsychiatric conditions—including ADHD and autism spectrum disorder—have a higher prevalence of sleep disturbances and lower adherence to behavioral programs (64). Lower educational attainment is an independent predictor of poor sleep quality and reduced adherence to sleep hygiene recommendations (160). Patients receiving antiseizure polytherapy, and those with limited social support networks, demonstrate consistently worse sleep quality and are more likely to require multimodal treatment strategies (160). These high-risk groups particularly benefit from systematic sleep disorder screening combined with personalized, multidisciplinary intervention planning (64, 146).

### 10.4 Melatonin and other chronobiological approaches

Melatonin may reduce sleep-onset latency and attenuate seizure severity, particularly as an adjunctive intervention in pharmacoresistant patients. However, the evidence base remains limited and of variable methodological quality. Recent meta-analyses report modest benefits on sleep latency and seizure severity, without significant effects on seizure frequency or health-related quality of life (161–163). Based on available data, melatonin may be considered as an adjunctive agent in patients with epilepsy and comorbid sleep initiation difficulties, but it should not be regarded as a substitute for optimized antiseizure pharmacotherapy (164). Beyond melatonin, chronobiological strategies—including light therapy for circadian phase resetting and structured timing of ASM administration aligned with individual seizure chronotypes—represent emerging therapeutic avenues that warrant further investigation.

## 11 Conclusion

The relationship between epilepsy and sleep is bidirectional and clinically significant. Epileptic activity disrupts sleep macro- and microarchitecture, impairing NREM and REM sleep with consequences on cognition, quality of life, and seizure control; conversely, sleep disorders lower the seizure threshold and contribute to disease progression. Improving sleep quality translates directly into better seizure control, making sleep disturbances a primary therapeutic target. Sleep stage exerts a clear modulatory role: NREM sleep facilitates the generation and propagation of interictal epileptiform discharges and seizures through thalamocortical synchronization, whereas REM sleep is protective for both ictal and interictal activity. This sleep-stage dependency carries direct implications for EEG interpretation and for understanding the nocturnal distribution of seizures. From a clinical perspective, systematic screening for sleep disturbances is strongly recommended in all patients with epilepsy, using validated questionnaires as a first step and polysomnography where indicated. Sleep deprivation EEG has no role in the differential diagnosis between epilepsy and parasomnias—an area fraught with diagnostic pitfalls given the substantial clinical overlap between NREM parasomnias, REM behavior disorder, and sleep-related epilepsies. Video-PSG is the indispensable tool, integrating sleep stage, motor semiology, and electrographic data to reach a reliable diagnosis. The choice of ASMs should also consider their differential effects on sleep architecture as these may either compound or mitigate sleep-related morbidity, and behavioral or chronobiological interventions represent valuable adjunctive strategies. In conclusion, an integrated, multidisciplinary approach addressing the sleep–epilepsy interface has the potential to improve seizure outcomes, preserve cognitive function, and enhance patients’ quality of life—translating this evidence into routine clinical practice remains both a priority and an opportunity.
